# Tryptase and tumor angiogenesis

**DOI:** 10.3389/fonc.2024.1500482

**Published:** 2024-12-19

**Authors:** Domenico Ribatti

**Affiliations:** Department of Translational Biomedicine and Neuroscience, University of Bari Medical School, Bari, Italy

**Keywords:** angiogenesis, mast cells, tryptase, tumor growth, proteases

## Abstract

Tryptases represent the most abundant constituent of human mast cells, involved in extracellular matrix degradation, contributing to wound healing and metastasis. Moreover, most recently, it has been demonstrated that tryptase is angiogenic both *in vitro* and *in vivo*. Tryptase-positive mast cell number increases parallelly with increased microvascular density in both solid and hematological tumors. The objective and the scope of this review article are to emphasize the important role of tryptase as one of the principal effectors of tumor angiogenesis mediated by mast cells. In this context, tryptase inhibitors may be considered a novel therapeutic approach in cancer treatment.

## Introduction

Angiogenesis, forming new blood vessels from pre-existing ones, occurs in various physiological and pathological conditions, such as embryonic development, wound healing, the menstrual cycle, and chronic inflammation and tumors. Tumor angiogenesis is linked to a switch in the equilibrium between positive and negative regulators and mainly depends on the release by neoplastic cells of growth factors specific to endothelial cells and it can stimulate the growth of host’s blood vessels (1, 79). Moreover, different classes of proteases, including matrix metalloproteinases (MMPs) (2), serine proteases (3), aminopeptidases (4), transmembrane proteases (TPs) (5), type II transmembrane serine proteases (TTSPs) (6), kallikrein-related peptidases (KLKs) (7), are involved in tumor angiogenesis (8).

Immune cells are also able to synthesize and secrete pro-angiogenic factors that promote tumor angiogenesis. Among these cells, mast cells exert both anti-tumorigenic and pro-tumorigenic roles. These cells produce several angiogenic factors, including fibroblast growth factor-2 (FGF-2), vascular endothelial growth factors (VEGF), and interleukin-8 (IL-8), as well as proteases, promoting tumor neovascularization. By contrast, mast cells inhibit tumor growth releasing cytokines and growth factors, including tumor necrosis factor-alpha (TNF-α), transforming growth factor beta (TGF-β), interferon-alpha (IFN-α), and bioactive monoamines (9).

An increased number of mast cells have been demonstrated in angiogenesis associated with vascular tumors, like hemangioma and hemangioblastoma, as well as several hematological and solid tumors, including lymphomas, multiple myeloma myelodysplastic syndrome, B-cell chronic lymphocytic leukemia, breast cancer, gastric and colorectal cancer, uterine cervix cancer, melanoma, and pulmonary adenocarcinoma, in which mast cell accumulation correlate with increased neovascularization, tumor aggressiveness, and poor prognosis (10). Conversely, mast cells have been demonstrated to play a protective role in the early stages of intestinal tumorigenesis (11). In this context, similarly to neutrophils (N1 and N2 subpopulations), and macrophages (M1 and M2 subpopulations), also mast cells are polarized toward anti-tumorigenic (MC1) or pro-tumorigenic (MC2) cell types (12).

Mast cells produce different biological mediators, including histamine, proteoglycans, proteases, cytokines, lipid mediators, and growth factors. Most proteins synthesized in mast cells are proteases, stored fully active in a complex with heparin (13). Mast cells are a reservoir of neutral proteases, packed in large amounts in the secretory granules, including tryptases, chymases, cathepsin C and G, and carboxypeptidase A3 (14). The secretion of these mediators is a consequence of mast cell degranulation and occurs as a response to physical factors, toxins, venoms, proteins, tissue proteases, and immune mechanisms, dependent or not dependent on IgE. Human tryptase is considered specific to mast cells (15), even if basophils contain and release tryptase (16).

Genetic analysis of tryptases in different species suggests that these proteases proliferated and changed rapidly during mammalian evolution, arising from ancestral membrane-anchored peptidases, which are present in a variety of vertebrate genomes such as reptiles, amphibians, and fish (17).

Tryptases, a group of 130 kD serine peptidases representing the most abundant constituent of human mast cells, are involved in extracellular matrix degradation, contributing to wound healing and metastasis. In humans, there are five isoforms of mast cell tryptase, α-, β-, γ-, δ-, and ε-tryptase. (18). Alpha-tryptases are classified in α-I and α-II tryptases, while β-tryptases are classified in β-I, β-II, and β-III tryptases (19). Alpha- and β-tryptases are the most abundant and clinically relevant, with approximately 90% sequence homology between them. Tryptases cleave fibronectin and type VI collagen, pro-enzyme forms of MMPs, and urokinase plasminogen activator (uPA), different bronchial and intestinal neuropeptides, such as calcitonin gene-related peptide (CGRP) and vasointestinal peptide (VIP), and IgE molecules, downregulating the allergic response.

βII-tryptase is stored in the secretory granules of mast cells. In contrast, a-pro-tryptase is secreted constitutively from mast cells as an inactive proenzyme.

The activation of βII-pro-tryptase involves two proteolytic steps. The most common mutations of the tryptase gene lead to loss of membrane anchoring, defective zymogen activation, or loss of catalytic function, thereby giving rise to changes in specificity (20). Extra copy numbers of tryptase α1 (TPSAB1) reflecting hereditary α-tryptasemia (HαT), a common genetic trait with increased copy numbers of the α-tryptase encoding gene, that correlates with mast cell activation-related events. In patients with mastocytosis, the presence of a HαT was associated with high serum tryptase independent of the clonal mast cell burden (21).

Tryptase plays a crucial role in mast cell ontogeny (22). The first source of mast cells is the extraembryonic yolk sac, on embryonic day 7. Mast cell progenitors circulate and enter peripheral tissues where they complete their differentiation, and embryonic mast cell populations are gradually replaced by definitive stem cell-derived progenitor cells. Transcriptome analysis of mast cells derived from human umbilical cord blood and peripheral blood, revealed a series of mast cell-specific genes, including tryptase α1 and β1, L-histidine decarboxylase, cathepsin G, and carboxypeptidase A (23).

In humans, mast cells containing tryptase only are designated as MCsT or “immune cells associated” mast cells predominantly located in the respiratory and intestinal mucosa, where they co-localize with T cells. Instead, mast cells that contain both tryptase and chymase referred to as MCsTC, are predominantly found in the skin, submucosa of the stomach and intestine, breast parenchyma, myocardium, lymph nodes, conjunctiva, and synovium (24, 78).

Secretion of tryptase from mast cells triggers the release of more tryptase from neighboring mast cells (25). Tryptase stimulates the proliferation of airway muscle cells (26), fibroblast migration and proliferation (27), and induces the synthesis and release of collagen from fibroblasts *in vitro* (28). In breast cancer, mast cell tryptase promotes myofibroblast differentiation in the tumor stroma (29). Moreover, tryptase stimulates vascular permeability and chemotaxis of neutrophils and eosinophils.

Tryptase levels in biological fluids reflect the number of mast cells (30). In healthy individuals, serum baseline tryptase levels are very stable over time, ranging between 1 and 15 ng/ml, while altered levels indicate the risk of severe allergic manifestations (31). The predominant indication for tryptase measurement is to document systemic mast cell activation conditions during anaphylaxis or episodes of mast cell activation syndromes. Serum tryptase has been described as a circulating predictive surrogate marker in colorectal cancer and in breast cancer before and after surgical resection when tryptase levels significantly decrease (32, 33).

This review article’s objective and scope are to emphasize the important role of tryptase as one of the principal effectors of tumor angiogenesis mediated by mast cells. In this context, tryptase inhibitors may be considered a novel therapeutic approach in cancer treatment.

## Tryptase and tumor angiogenesis

Paul Ehrlich discussed the possible connection of mast cells with tumor growth and progression early in 1878 (34). Indeed, most tumors contain inflammatory cell infiltrates, which often include mast cells. Thus, the importance of a potential functional link between chronic inflammation and cancer has long been recognized and the question of the possible contribution of mast cells to tumor development has progressively emerged. It was Rudolf Virchow in 1863, who critically recognized the presence of inflammatory cells infiltrating neoplastic tissues and first established a causative connection between the “lymphoreticular infiltrate” at sites of chronic inflammation and cancer (35).

There is evidence that different cell types of the innate immune system, including mast cells and macrophages play a critical role in enhancing tumor angiogenesis, either directly through the release of angiogenic cytokines and proteolytic enzymes, or indirectly through paracrine signals.

It is still not completely known and clarified the role of tryptase as another pro-angiogenic factor released by mast cells. Tryptase-positive mast cells are among the first immune cells recruited to tumor sites in response to the chemotactic stimuli and are increased in solid tumors, at the boundary between malignant and healthy tissues (29, 36). The main chemoattractant factor produced by tumor cells is stem cell factor (SCF), the ligand for the tyrosine kinase receptor kit (CD 117) expressed by mast cells and considered the most important factor involved in the regulation of mast cell number in physiological conditions (37, 38).

Blair et al. (39) for the first time investigated *in vitro* the angiogenic potential of tryptase demonstrating that tryptase added to microvascular endothelial cells cultured on Matrigel causes an increase in capillary growth in a dose-dependent fashion, and specific tryptase inhibitors suppressed this effect.

Tryptase stimulates endothelial cell release of IL-1, IL-6, IL-8, SCF, TNF-α, and other inflammatory mediators (40). Moreover, tryptase promotes chemotaxis of neutrophils and macrophages, which induces the new formation of capillaries, and activates MMP-9, which favors the release of angiogenic factors stored in the extracellular matrix (41).

**Figure 1:**
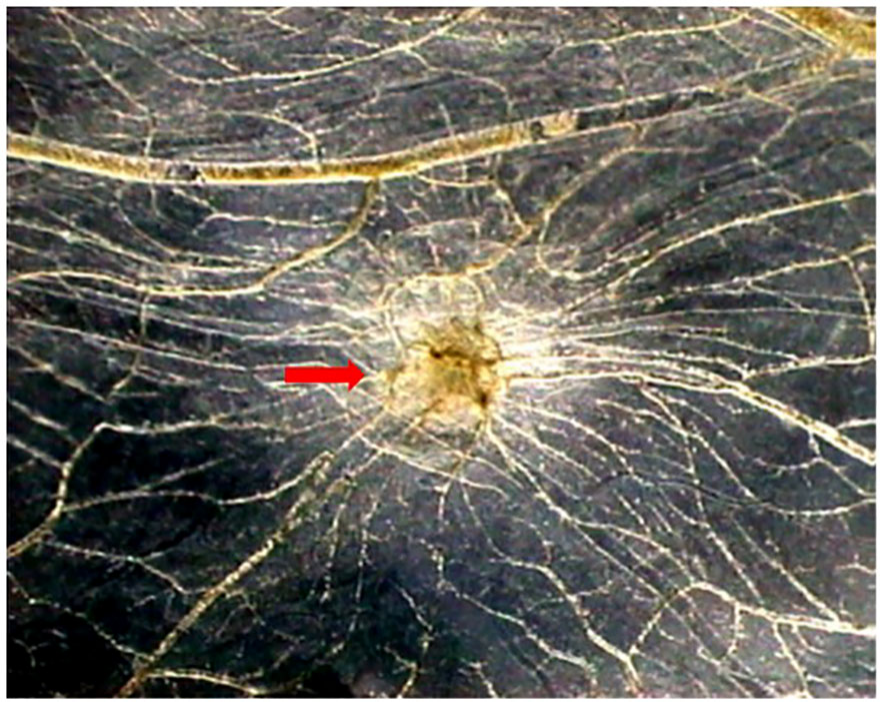
Tryptase is angiogenic *in vivo* in the CAM assay. Macroscopic picture of a CAM at day 12 of incubation treated with tryptase. Note the presence of numerous blood vessels converging toward the implant (arrow) (Modified from 42).

We have demonstrated an angiogenic activity of human recombinant tryptase with a grade of purity of 95% *in vivo* in the chick embryo chorioallantoic membrane (CAM) assay (**Figure 1**), comparable to the angiogenic response induced by a well-known angiogenic cytokine, namely VEGF (42). Tryptase contributes to atherosclerotic plaque angiogenesis and hemorrhage by regulating VEGF, PA inhibitor (PAI), and tissue PA (tPA) expression (43).

Tryptase is documented to play a role in tumor angiogenesis (**Table 1**). Elevation of different angiogenic factors, including VEGF, FGF-2, and platelet-derived growth factor (PDGF) in response to tryptase plays a key role in tumor angiogenesis (10). Tryptase acts on the proteinase-activated receptor-2 (PAR-2), stimulating tumor angiogenesis (44), and induces PAR-2. Tryptase induces PAR-2 proliferative effects on a human colon carcinoma cell line (45). PAR-2 leads to the release of IL-6 and granulocyte-macrophage colony-stimulating factor (GM-CSF), acting as angiogenic factors (46).

**Table 1:** Different roles of tryptase in tumor angiogenesis.

Increased levels of different angiogenic factors (10)
Activation of PAR-2 (44, 45)
Release of IL-6 and GM-CSF (46)
Increased microvascular density in different solid human cancers (32, 47–55, 56–63).
Increased microvascular density in different hematological human cancers (64–68).

Tryptase-positive mast cell number increases parallelly with increased microvascular density in solid tumors, including malignant melanoma (47, 48), endometrial carcinoma (49, 53, 54), breast cancer (50, 56, 57), uterine leiomyomas (51), gastric cancer (52, 55, 58), colorectal cancer (61, 62), pancreatic ductal adenocarcinoma (59, 60, 63). In hematological tumors tryptase-positive mast cell count correlates with angiogenesis in multiple myeloma (64), in B cell non-Hodgkin’s lymphomas (65), in myelodysplastic syndrome (66) and B cell chronic lymphocytic leukemia (67, 68).

## Therapeutic implications

**Figure 2:**
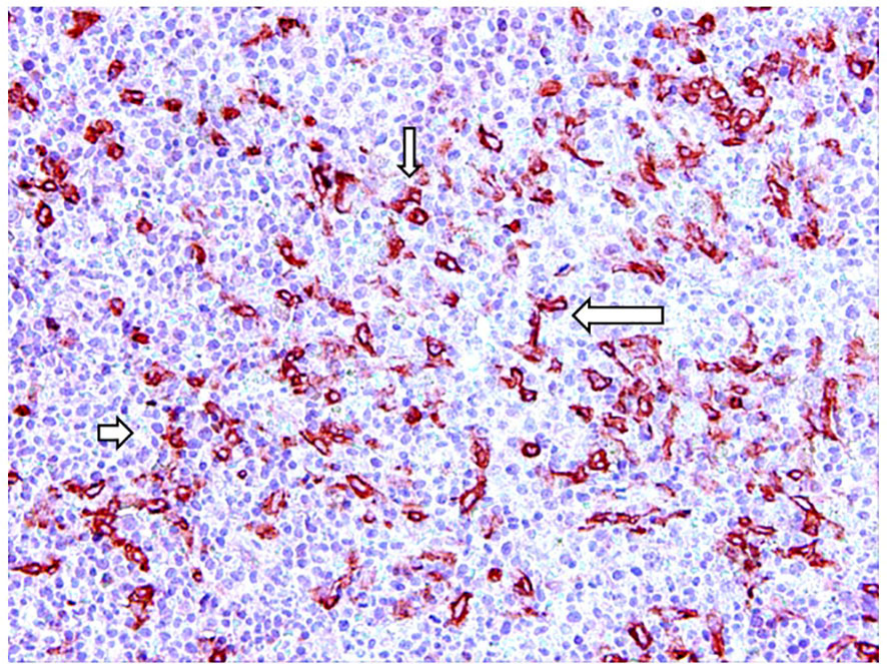
Tryptase-positive mast cells (arrows) in red in a bioptic specimen of human skin mastocytosis.

Reducing mast cell number is a therapeutic approach in mastocytosis, characterized by mast cell accumulation in the skin (**Figure 2**) and other tissues, and other diseases characterized by an increase in mast cell number. Different pharmacological agents such as omalizumab, imatinib, disodium cromoglycate, H1 receptors antagonists, steroids, and non-steroidal anti-inflammatory drugs have been developed to modulate the functions of mast cells. The interplay between mast cells and tumor angiogenesis suggests considering the therapeutic use of inhibitors, which specifically target the angiogenic activity of tryptase. Cromolyn, an inhibitor of mast cell degranulation, reduces the expansion and survival of pancreatic cancer and endothelial cells (69). The combination of cromolyn with anti-angiogenic therapy increases the therapeutic efficacy (70). Tryptase inhibitors such as gabexate mesilate and nafamostat mesilate, two inhibitors of trypsin-like serine proteases (71–73) might be used as anti-angiogenic-agents through tryptase inhibition in combination with chemotherapy in the treatment of cancer (**Table 2**). Anti-angiogenic activity of gabexate mesilate in colon and pancreatic cancer may be due to a selective inhibition of mast cell tryptase (73–75). Nafamostat mesilate inhibits the tryptase-induced proliferation of tumor cells (45). Nafamostat exerts anti-angiogenic activity in pancreatic cancer through blockade of nuclear factor kappa-B (NF-kB) activation, which is mediated by tryptase through PAR-2 (76). Pancreatic cancer cell lines injected into nude mice with tryptase induced the formation of tumors larger than those developed in non-treated mice and nafamostat suppressed the tumorigenic effect of tryptase (77). Finally, different anti-cancer agents including sorafenib, sunitinib, pazopanib, axitinib, and masitinib, are all targeted against c-KitR, whose activation leads to the release of tryptase by mast cells.

**Table 2:** Tryptase inhibitors as anti-cancer agents.

Gabexate mesilate, colon cancer (74)
Gabexate mesilate, colon cancer (75)
Gabexate mesilate, pancreatic cancer (73)
Nafomostat mesilate, colon cancer (45)
Nafomostat mesilate, pancreatic cancer (77)

## Concluding remarks

It is well established that mast cell accumulation accompanies most malignancies. However, the knowledge of how mast cells functionally impact tumors is still under investigation. Mast cells modulate the biological activity of immune and non-immune components of the tumor microenvironment through the release of a plethora of mediators, including tryptase. In this context, despite the critical role of tryptase in tumor growth and angiogenesis, the development of effective inhibitors has been a complex challenge, mainly due to the intricate mechanisms governing its activation and regulation. Although numerous tryptase inhibitors have been previously reported, it is important to note that these are largely in investigational stages and have not yet received FDA approval. It is conceivable that tryptase inhibitors might be combined with other novel anticancer approaches, such as anti-PD-1/PDL-1 therapy. The blockade of the PD-1/PD-L1 interaction has been suggested as a useful and novel therapeutic approach in the treatment of tumors in which mast cells are involved.
